# The association between dietary glycemic index and cardio-metabolic risk factors in obese individuals

**DOI:** 10.1186/s40795-022-00608-6

**Published:** 2022-10-17

**Authors:** Goli Siri, Mahsa Mahmoudinezhad, Samira Alesaeidi, Mahdieh Abbasalizad Farhangi, Abnoos Mokhtari Ardekani

**Affiliations:** 1 Department of Internal Medicine, Amir Alam Hospital, Tehran University of Medical Sciences, Tehran, Iran; 2 Department of Community Nutrition, Faculty of Nutrition, Tabriz University of Medical Sciences, Tabriz, Iran; 3 Department of Internal Medicine and Rheumatology, Rheumatology Research Center, Tehran University of Medical Sciences, Tehran, Iran; 4 Endocrinology and Metabolism Research Center, Institute of Basic and Clinical Physiology Sciences, & Physiology Research Center, Kerman University of Medical Sciences, Kerman, Iran; 5 Drug Applied Research Center, Tabriz University of Medical Sciences, Tabriz, Iran

**Keywords:** Fatty acid desaturase 2, Glycemic index, Metabolic parameters, Obesity

## Abstract

**Background:**

The dietary glycemic index (GI) has been introduced as a novel index to elucidate the potential of foods to increase postprandial glucose. According to the limited available data about the association of GI with cardio-metabolic risk factors such as lipid profile, blood glucose markers, and blood pressure in developing countries, the current study was conducted to investigate this association in apparently obese individuals.

**Method and material:**

Three hundred forty-seven obese adults were recruited in the present cross-sectional study. A validated 147-food item semi-quantitative food frequency questionnaire (FFQ) was used to evaluate the usual dietary intake of study participants. Dietary GI was calculated using the international GI database. Fatty acid desaturase (FADs)2 gene variants were determined according to polymerase chain reaction-restriction fragment length polymorphism (PCR-RFLP). ANOVA was used to compare study variables across different tertile of GI.

**Results:**

We found significant differences in terms of anthropometric parameters [weight (P = 0.038), waist circumference (WC) (P = 0.023), weight to hip ratio (WHR) (P = 0.007), and fat-free mass (FFM) (P < 0.001)] between different tertiles of GI. Similarly, energy and macronutrient intakes had a significant difference across dietary GI, and subjects with a higher dietary intake of energy and macronutrients (carbohydrate, protein, and total fat) were assigned to the third tertile of dietary GI (P < 0.001). While there was no significant difference in terms of cardio-metabolic risk factors in different dietary GI tertiles. Moreover, the total GI score was non-significantly higher in the TT genotype of FADS2 gene polymorphism compared with other genotypes. While no significant difference was observed between FADS2 genotype frequencies in different GI tertiles.

**Conclusion:**

Calculated dietary GI was associated with several cardio-metabolic risk factors in obese individuals. However, further prospective studies and clinical trials are needed to confirm our findings.

## Background

Non-communicable diseases (NCDs), as a global emergency, impose a large economic burden on society and contribute to a greater rate of comorbidities worldwide [1, 2]. NCDs occur following cardio-metabolic disorders [3]. Cardio-metabolic disorders are a set of pathophysiological conditions which result from a disturbance in normal metabolism [4]. Cardio-metabolic risk factors enhance the possibilities of obesity, metabolic syndrome, hypertension, type 2 diabetes, ischemic heart disease, and stroke [5–7]. Since cardio-metabolic diseases are present long before they become apparent and they have reached epidemic status worldwide, it is critical to notify different preventive strategies including dietary behaviors and physical activity to modify their prevalence [8–11]. Diet as a modifiable risk factor and first-line therapy drives a major impact on cardio-metabolic risk factors. Recently, there is a growing interest in the association between dietary glycemic index (GI) and cardio-metabolic risk factors. The type and amount of dietary carbohydrates are known as the main determinants of postprandial blood glucose and insulin levels [12–15]. The GI is an index of ranking dietary foods according to their potential to increase postprandial glucose [12]. The GI presents the effects of the carbohydrate portion on postprandial glucose compared with white bread or oral glucose ingestion as a reference food [24–27]. Increased level of postprandial glucose affects oxidative stress status and plays a critical role in the generation of endothelial and cardiovascular diseases [16–18]. Regarding the higher consumption of carbohydrates as the main source of energy in developing countries, GI seems to be a good indicator of cardio-metabolic risk factors [19]. Despite the several studies conducted about relation to GI and the risk of cardio-metabolic diseases, the results are inconsistent. In a cross-sectional study in Japan, dietary GI was positively associated with body mass index (BMI) in female farmers with traditional dietary habits [20]. In contrast, Du et al. demonstrated that GI level is not associated with weight changes; while dietary GI was positively associated with changes in waist circumference (WC) [21]. Moreover, previous studies indicated that high dietary GI may contribute to dietary-associated cardio-metabolic risk factors [22]. Both Ma X. and Mirrahimi A. et al. reported that GI is in a direct association with cardio-metabolic risk factors such as high-density lipoprotein cholesterol (HDL-C) [23, 24]. Moreover, from the personalized nutrition aspect, genome-wide association studies (GWAS) have revealed the role of several single nucleotide polymorphisms (SNPs) to affect cardiometabolic risk factors. In this regard, fatty acid desaturase 2 (FADS2) gene rs174583 polymorphism that is mostly involved in fatty acids metabolism [25] is reported to affect carbohydrate metabolism and that high dietary carbohydrate might increase its gene expression [26]. FADS2 is an endoplasmic reticulum membrane-bound protein located on chromosome 11 (11q12–13.1), which is comprised of 12 exons and 11 introns [27–30]. Previous studies have suggested that dietary intakes may interact with FADS2 gene rs174583 polymorphism to modify cardiometabolic factors in obese individuals [8, 27, 31]. In the study by Drag J et al. [26], high dietary carbohydrate intake was associated with increased FADS2 gene expression. Also, in another study by Muzsik A et al. [32], FA concentrations in red blood cells (RBCs), as an indicator of FADS2 polymorphism, was affected by dietary carbohydrate intake among postmenopausal women. As indicated in the previous studies, the Iranian population tended to receive more than 60% of their energy intake from carbohydrates (especially from high GI foods such as refined grains). So their obesity patterns seem to be different from other developed countries [19]. On the other hand, there are limited data about the association between dietary GI and the risk of cardio-metabolic diseases in developing countries. Moreover, there is almost no study to evaluate the association between FADS2 gene polymorphism and dietary GI in the apparently healthy obese populations. Likewise, the present study was conducted to address this knowledge gap.

## Methods and materials

### Participants

In the present cross-sectional study, 347 healthy obese individuals were enrolled. Convenience sampling was used to recruit participants. The participants were recruited from the combination of two previous projects among obese individuals [33–35]. Study subjects were invited by public announcements and were included if they met inclusion criteria of age between 20 and 50 years old and being obese (BMI > 30 kg/m^2^). Participants were recruited from both genders. Exclusion criteria were as follows: any history of chronic diseases (e.g., diabetes, hepatic disorders, renal diseases, cardiovascular diseases, hypertension, hyperlipidemia, and cancer), pregnancy, lactation, and using any medications affecting weight. Written consent was obtained from all of the participants. The ethical committee of the Tabriz University of Medical Sciences approved the study protocol (registration codes: IR.TBZMED.REC.1399.062 and IR.TBZMED.REC.1400.454).

### Demographic, anthropometric, and blood pressure assessments

A trained interviewer completed the questionnaire on socioeconomic status (SES) and demographic information. The SES was including information about family size, occupation, educational status, and house ownership. Also, other information such as age, sex, marital status, and medical history for each participant were completed to assess demographic status. The International Physical Activity Questionnaire (IPAQ) was used to estimate physical activity level [36]. Accordingly, physical activity levels of below 600, between 600 and 3000, and higher than 3000 METs-min/week were considered as low, moderate, and high levels of physical activity respectively. Weight and height were measured using a Seca scale (Seca, Germany) with a precision of 100 g and a tape with a precision of 0.1 cm, respectively. WC was measured using a flexible tape to the nearest 0.1 cm, at the narrowest level without applying any pressure to the body. Bioelectrical impedance analysis (BIA) technology (Tanita, BC-418 MA, Tokyo, Japan) was used to determine the body composition. Blood pressure (BP) measurements were conducted using a standard mercury sphygmomanometer (Rudolf Riester GmbH, Jungingen, Germany).

### Biochemical assessments

Biochemical parameters were evaluated in 10 ml blood samples that were taken from each participant. Blood samples were centrifuged (at 4◦C and 4500 rpm for 10 min) to separate sera and plasma. Serum fasting glucose (FSG), triglyceride (TG), total cholesterol (TC), and HDL-C were assayed using commercial kits (Pars Azmoon, Tehran, Iran). Low-density lipoprotein cholesterol (LDL) concentration was calculated using the Friedewald Eq. [37– 39]. Moreover, serum insulin level was analyzed with commercially available enzyme-linked immunosorbent assay kits (ELISA; Bioassay Technology Laboratory, Shanghai Korean Biotech, Shanghai City, China) based on the manufacturer’s instructions. In addition, the indicators of insulin resistance [homeostasis model assessment-insulin resistance index (HOMA-IR) and quantitative insulin sensitivity check index (QUICKI)] were calculated based on the standard formula as follows [40, 41]:

HOMA-IR = [FSG (mg/dl) * fasting insulin (µU/ml)]/ 405.

QUICKI = 1/ (log (fasting insulin µU/ml) + log (fasting glucose mg/dl)).

### Dietary assessments

All individuals completed a validated and interviewer-administered 168- item food frequency questionnaire (FFQ) with the mean energy-adjusted reliability coefficients (alpha Cronbach) of 0.39–0.79 [42, 43]. The individuals were asked to report their usual dietary intakes during the past 12 months based on standard serving sized and all records were converted to gram/day using the handbook of household measures [44]. Energy and nutrients intakes were estimated using Nutritionist IV software. We used the following formula according to the international table of GI values with the optimal match to obtain the total dietary GI [45]:

Ʃ (GI*available carbohydrate of food)/ Total available carbohydrate.

Some food items were not available in this database and we used chemically-similar food items instead. Some traditional sweets were considered sugar. Oral glucose was considered as the reference food in all GI values. The dietary glycemic load (GL) was calculated as (total GI/ total available carbohydrate)/100 [46].

### Genotyping

Blood samples were collected from all individuals and the chloroform technique was used to extract genomic DNA. All DNAs were expected to be genotyped for rs174583. SNP rs174583 was genotyped by the polymerase chain reaction-restriction fragment length polymorphism (PCR-RFLP) method. The PCR was done using primers with the following sequence: forward, 5′ AGGAAGCAGACCACAGAGTC 3′; reverse, 5′ TC CTTCGTCTGGTGTCTCAG 3′. PCR reactions contained: 5 µl Master Mix (Ampliqon; Denmark), 2 µl extracted DNA, 1 µl primers and 2 µl distilled water. TauI (cat. num. ER1652, USA) as a restriction enzyme was used to digest amplified DNA due to the restriction sites on the sequence of the amplified DNA. The DNA thermocycler (BIO-RAD T100 Thermal Cycler), that was used for PCR cycling, was set-out according to the following command: 95 °C for 10 min of denaturation, amplification consisted of 35 cycles at 94 °C, annealing at 60 °C for 20 s and 50 s of extension at 74 °C and final extension occurred at 74 °C for 10 min. Fragments containing three possible genotypes of the FADS2 rs2239670 were detected: TT, TC, and CC.

### Sample size calculation

The sample size of the current study was calculated based on Hu LT et al.’s study [47] using G-power software, considering α = 0.05, β = 0.20, and power of 80% and the correlation equation of r = 0.25. Accordingly, the calculated sample size was estimated as 310 individuals. Considering the drop-out rate of 12%, a total of 347 individuals was sufficient to accomplish the study.

### Statistical analysis

Statistical analysis was conducted using SPSS (SPSS, Inc., Chicago, IL, version 21). The distribution of variables was tested for normality. Normally distributed and non-normal variables were reported as means ± standard deviations (SD) and median (min, max), respectively. Categorical variables were reported as frequencies or percentages. One-way analysis of variance (ANOVA) and chi-square tests were used to compare continuous and discrete variables across different categories respectively. Also, to compare variables with non-normal distribution across different GI categories, Kruskal-Wallis test was used. The P-values less than 0.05 were considered statistically significant.

## Results

The socio-demographic and anthropometric characteristics of the study participants are summarized in Table 1. In the current study, 58.2% of the participants were men. The mean age of included individuals was 38.08 years old and the mean ± SD of the weight of participants was 92.11 ± 14.44. Approximately, 50% of the study participants had a low physical activity with middle SES. Individuals with a higher SES level were assigned to the third tertile of GI. According to the comparison of anthropometric variables between different dietary GI categories, adults consuming higher dietary GI tended to show significantly higher weight (P = 0.038) and WC (P = 0.023) compared to other groups. It has been shown that WHR was significantly different between tertiles of dietary GI (P = 0.007). The comparison of daily macronutrients and several food groups’ intake in different dietary GI tertiles is presented in Table 2. The mean ± SD of energy intake was 3016.71 ± 1094 kcal/day. Dietary intake of energy, macronutrient, and fibers were significantly different across GI categories (P < 0.001). Subjects with a higher intake of energy and macronutrients (carbohydrate, protein, and total fat) were assigned to the third tertile of dietary GI. In addition, dietary intake of grains, egg, red meat, and dairy products was significantly different across dietary GI tertiles (P < 0.05). Biochemical variables across different dietary GI tertiles are presented in Table 3. Those at the top tertile of the dietary GI were younger and were more likely to consume high carbohydrate-containing foods compared with the lowest tertile (P < 0.001). Likewise, they showed a non-significantly higher level of serum glucose level (P = 0.325). The overall prevalence of rs174583 genotypes was 37.8, 51.9, and 10.3 for CC, CT, and TT, respectively. Moreover, there was a non-significant difference between FADS2 rs174583 variants in terms of GI score (Table 4; P > 0.05).

**Table 1:** Comparison of demographic and anthropometric characteristics between different dietary GI tertiles

Variables	GI tertiles			
	**1st tertile**	**2nd tertile**	**3rd tertile**	**P-value**
**Age (year)**	41.98 ± 9.63	40.15 ± 8.97	39.55 ± 8.47	0.112
**Sex, man (%)**	23.6	36.4	40	0.121
**Weight (kg)**	89.31 ± 15.26	93.93 ± 15.13	93.09 ± 12.71	**0.038**
**Height (cm)**	166.13 ± 10.19	168.80 ± 9.92	168.95 ± 9.36	0.054
**FFM (kg)**	56.34 ± 11.74	65.29 ± 12.06	64.13 ± 11.68	**< 0.001**
**WC (cm)**	104.70 ± 9.28	107.91 ± 10.49	107.56 ± 8.89	**0.023**
**WHR**	0.91 ± 0.08	0.93 ± 0.07	0.95 ± 0.06	**0.007**
**BMI (kg/m** ^**2**^ **)**	32.36 ± 4.94	32.98 ± 5.44	32.62 ± 4.07	0.631
**Physical activity (%)**				0.310
***Low***	28.9	30	41.1	
***Moderate***	36.5	32.7	30.8	
***High***	19.6	30.4	50	
**SES, (%)**				0.509
**Low**	40	20	40	
**Middle**	33.3	27.3	39.4	
**High**	22.9	36.1	41	
**FADS2 genotype, (%)**				0.205
**TT**	18.8	25	56.2	
**TC**	23.5	37	39.5	
**CC**	35.6	22	42.4	

GI, Glycemic index; FFM; Fat free mass, WC; Waist circumference, WHR; Waist to hip ratio, BMI; Body mass index, SES; Socio-economic status, FADS2; Fatty acid desaturase 2; Data are mean ± standard deviation (SD); P-value obtained from ANOVA or chi-square test

**Table 2:** Comparison of dietary macronutrient intakes and food groups between different dietary GI tertiles

Variables	GI tertiles			
	**1st tertile**	**2nd tertile**	**3rd tertile**	**P-value**
**Energy (kcal/day)**	2233.56 ± 590.46	3037.47 ± 783.69	3771 ± 1215.61	**< 0.001**
**Nutrients**				
***Carbohydrate (g/day)***	336.47 ± 114.42	448.97 ± 115.17	567.31 ± 183.70	**< 0.001**
***Protein (g/day)***	76.43 ± 19.17	102.27 ± 31.27	120.04 ± 42.90	**< 0.001**
***Total fat (g/day)***	73.54 ± 25.92	102.31 ± 40.84	125.60 ± 54.78	**< 0.001**
***Dietary fiber (g/day)***	46.66 ± 18.08	65.84 ± 32.96	85.55 ± 48.25	**< 0.001**
**Food groups**				
***Grains (g/day)***	8.5 (3.75, 18.1)	15.1 (4.89, 30.33)	17.62 (3.57, 42.49)	**< 0.001**
***Poultry (g/day)***	0.72 (0.09, 3.32)	0.81 (0.09, 3.24)	0.76 (0.001, 3.44)	0.696
***Egg (g/day)***	0.51 (0.01, 2.44)	0.71 (0.07, 2.14)	0.73 (0.04, 2.44)	**< 0.001**
***Red meats (g/day)***	15.32 ± 12.24	25.21 ± 28.35	24.92 ± 27.30	**0.008**
***Fish (g/day)***	10.66 ± 13.92	10.52 ± 15.56	9.41 ± 11.58	0.601
***Fruits (g/day)***	3.78 (0.22, 21.63)	3.48 (0.15,10.21)	4.95 (0.7, 9.60)	0.423
***Vegetables (g/day)***	3.17 (0.93, 9.89)	3.71 (0.75, 13.30)	4.46 (1.01, 15.26)	0.101
***Dairy products (g/day)***	1.74 (0.10, 4.78)	1.99 (0.39, 6.13)	2.37 (0.21, 7.44)	**0.029**
***Legumes (g/day)***	0.60 (0.05, 1.74)	0.72 (0.02, 3.75)	0.85 (0.13, 5.94)	0.099

GI, Glycemic index; Data are mean ± standard deviation (SD) and median (min, max) for normally and unmorally distributed data respectively. P-value obtained from ANOVA or Kruskal-wallis test

**Table 3:** Comparison of demographic and anthropometric characteristics between different dietary GI tertiles

Variables	GI tertiles			
	**1st tertile**	**2nd tertile**	**3rd tertile**	**P-value**
**TG (mg/ dl)**	144.55 ± 80.36	167.60 ± 122.02	140.76 ± 66.90	0.064
**TC (mg/ dl)**	196.24 ± 41.90	191.30 ± 34.29	187.74 ± 33.19	0.219
**LDL-C (mg/ dl)**	128.74 ± 36.61	121.53 ± 30.77	120.53 ± 27.49	0.110
**HDL-C (mg/ dl)**	44.66 ± 10.08	42.81 ± 9.94	43.10 ± 8.37	0.295
**FSG (mg/ dl)**	90.53 ± 11.90	93.38 ± 18.80	94.24 ± 25.10	0.325
**Insulin (U/mL)**	16.82 (1.50, 65.10)	15.35 (2.40, 35.30)	15.92 (1.80, 150.80)	0.205
**HOMA-IR**	3.88 (0.25, 18.49)	3.54 (0.48, 8.80)	3.77 (0.29, 32.02)	0.435
**QUICKI**	0.32 ± 0.04	0.32 ± 0.02	0.33 ± 0.03	0.592
**SBP (mmHg)**	125.46 ± 15.86	122.74 ± 13.76	120.01 ± 18.70	**0.043**
**DBP (mmHg)**	83.71 ± 11.44	81.35 ± 11.11	79.95 ± 12.38	**0.049**

GI, Glycemic index; TG; Triglyceride, TC; Total cholesterol, LDL-C; Low density lipoprotein cholesterol, HDL-C; High density lipoprotein cholesterol, FSG, Fasting serum glucose; HOMA; Homeostasis model assessment-insulin resistance index, QUICKI; Quantitative insulin sensitivity check index, SBP; Systolic blood pressure, DBP; Diastolic blood pressure. P-value obtained from ANOVA or Kruskal-Wallis test

**Table 4:** Comparison of dietary GI between different FADS2 rs174583 gene variants

	Genotype			
	**TT**	**TC**	**CC**	**P-value**
**Prevalence (%)**	10.3	51.9	37.8	
**GI index**	35.12 (13.58, 128.4)	24.45 (6.45, 77.36)	25.34 (9.69, 62.84)	0.190

GI, Glycemic index; FADS2, Fatty acid desaturase 2; P-value obtained from ANOVA or Kruskal-wallis test

## Discussion

**Fig. 1:**
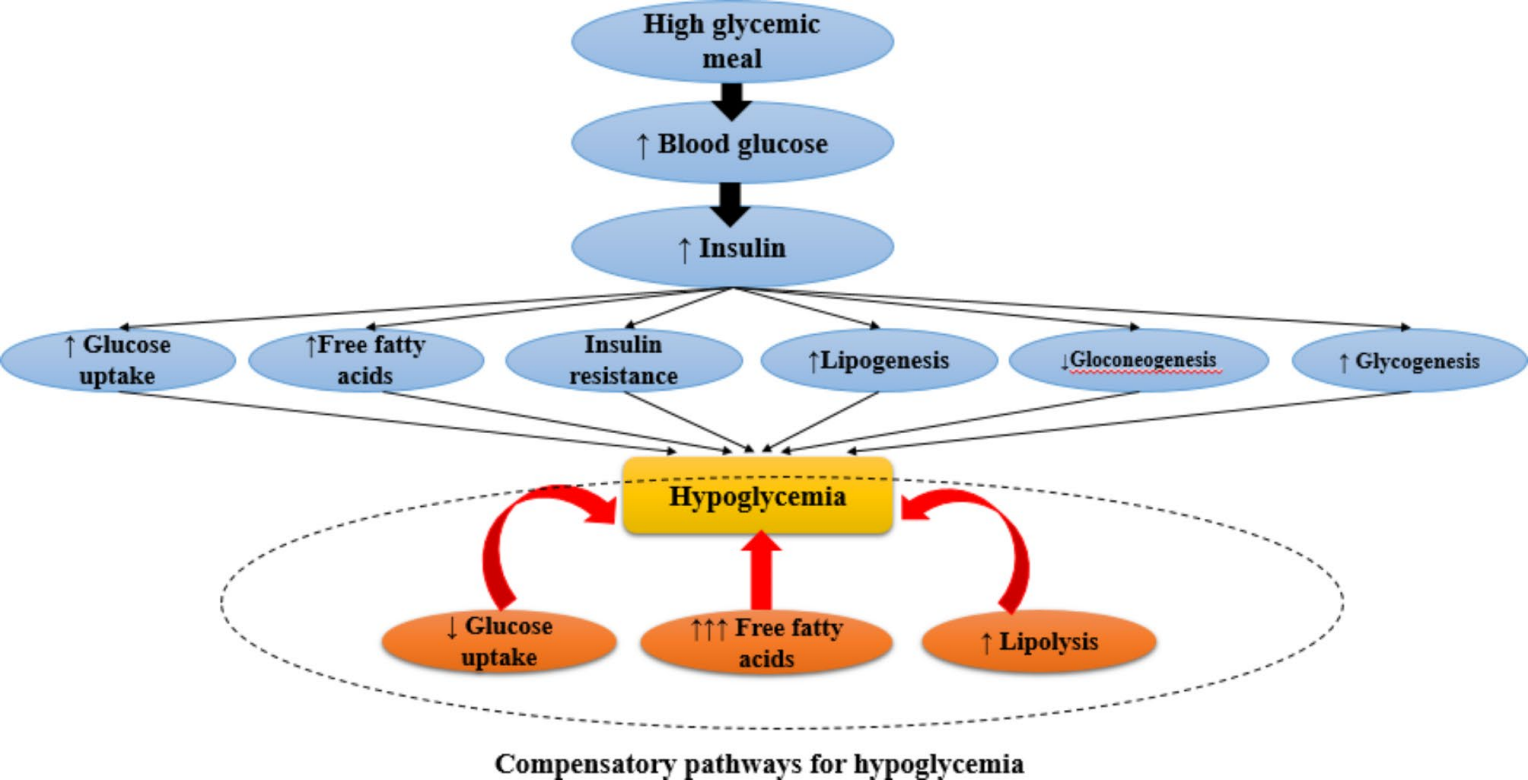
The effects of high-GI meal contributing to hypoglycemia and compensatory pathways to reduce hypoglycemia

To the best of our knowledge, this is the first study that evaluated the association between dietary GI and cardio-metabolic risk factors in the north-west population of Iran. Adults who consumed low-GI foods had significantly lower weight and WC. It seems that total carbohydrate intake and percentage of calories from carbohydrates may be associated with BMI. In agreement with our results, Ma et al. revealed that type of carbohydrate may affect body weight and GI was positively associated with BMI in their study [48]. In addition, a prospective cohort study which was conducted in five European countries showed that dietary GI is associated with WC [21]. Also, it has been shown that a high dietary GI is associated with greater odds of abdominal obesity in women in comparison to a low dietary GI [49]. On the other hand, previous studies indicated that high-GI foods play a role in nutrient partitioning and hunger which contribute to body fat storage [50]. Moreover, in an animal study, rats were fed with amylose (low GI starch) showed larger adipocyte diameter, increased Glut 4 gene expression in fat tissue, increased glucose incorporation to lipids, and developed obesity, compared to rats fed with amylopectin (high GI starch) [51]. Likewise, a systematic review and meta-analysis demonstrated that a low GI diet might be a practical method to prevent obesity and obesity-related comorbidities [52, 53]. However, some studies failed to find such associations that may be attributed to different study designs and populations [54]. Furthermore, subjects with a high intake of energy and macronutrients (carbohydrate, protein and, total fat) were assigned to the third tertile of dietary GI. In total, 60% of calorie intake in each tertile of GI was composed of carbohydrates approximately which is higher than in western diets. In addition, intake of the grains, egg, red meat, and dairy products showed a significant difference across tertile of dietary GI. Moreover, in the lowest tertile of GI, the highest concentration of HDL cholesterol was observed. Similarly, Amano et al. showed that the highest concentration of HDL cholesterol was attributed to the subjects consuming lower GI foods in Japanese women [55]. Also, the study of a nationally representative sample of US adults elucidated that high dietary GI is associated with lower concentration of HDL plasma too [56]. It suggests that dietary GI may be associated with the risk of coronary diseases in this way. Several observational studies have indicated that a decrease of 0.026 mmol/L in HDL-C level, increases the risk of coronary diseases by about 3.2% in women and 1.9–2.3% in men approximately [56]. In addition, adults adhering to high-GI foods in our study showed higher concentrations of serum glucose level. Buyken et al. demonstrated that a lower dietary GI is associated with lower HbA1c levels in type 1 diabetes mellitus among England population [57]. It seems that these effects of low-GI foods might be possible through decreased glucose levels and insulin responses that lead to increased satiety and decreased energy intake to prevent obesity [58]. Some supporting mechanisms in this regard that point to the association between dietary GI and cardio-metabolic risk factors are known. Prolonged hyperglycemia followed by an increase in insulin secretion may disturb pancreatic β-cell function and results in glucose intolerance and the development of diabetes consequently. Also, hyperglycemia affects free fatty acids levels directly. High-GI meal stimulates glycogenesis and lipogenesis then, an increased level of insulin in response to a high-GI meal causes hypoglycemia after 2–4 h. Afterward, late in the postprandial period, stimulation of glycogenolytic and gluconeogenic pathways and increased concentration of free fatty acids compensate for hypoglycemia [58] (Fig. 1). Moreover, the present study showed a lower and marginally significant level of DBP in the higher level of GI. Similarly, Sebely P. et al. found no significant changes among two study groups with the lower and higher levels of GI [59]. Although in the current study we did not observe any difference in FADS2 genotype frequency between GI tertiles; however, GI in TT genotype of FADS2 rs174583 gene variants was non-significantly higher than other genotypes. Previous studies also revealed that the TT genotype of FADS2 gene variants is considered as a risk allele in different populations; Mazoochian et al. reported the highest levels of cardio-metabolic parameters among homozygotes for T alleles among patients with type 2 diabetes mellitus in Iran [60]. Moreover, similar findings were observed in a study in South Korea [61]. Our results were also in agreement with another study conducted among Japanese males, reporting a higher risk of metabolic abnormalities in homozygotes for the T allele of FADS2 gene rs174583 polymorphism [62]. Also, in another part of the current project, we observed a higher intake of advanced glycation end products (AGEs) intake in carriers of the TT genotype revealing adherence to unhealthy food intake in carriers of this genotype [63]. The current study is subject to some limitations; first, according to different dietary patterns, it is hard to generalize our findings to other populations. Second, the cross-sectional study design makes it difficult to understand causal relationships between dietary GI and cardio-metabolic risk factors. However, it is worth noting the strengths of the study too. The previous studies were conducted in a small sample size with different populations. Previous studies indicated that the Iranian population tended to receive more than 60% of their energy intake from carbohydrates. So their obesity patterns seem to be different from other developed countries. Moreover, the present study involved the effect of genetic susceptibility and considered a tri-polar perspective in terms of diet and gene interaction about the cardio-metabolic risk factors.

## Conclusion

Calculated dietary GI was associated with some cardio-metabolic risk factors in northwest population of Iran. Our results suggested that a high-GI diet might be associated with higher weight, WC, WHR, and energy intake in adults. However, further prospective studies and clinical trials are needed to confirm our findings.

## Data Availability

All of the data are available with reasonable request from the corresponding author.
